# Engineered miR-214 enriched Schwann cell-derived extracellular vesicles amplify therapeutic efficacy for peripheral neuropathy in T2D mice

**DOI:** 10.3389/fncel.2025.1649830

**Published:** 2025-08-29

**Authors:** Lei Wang, Xuerong Lu, Alexandra Szalad, Yi Zhang, Yanfeng Li, Mei Lu, Amy Kemper, Zhongwu Liu, Xian Shuang Liu, Michael Chopp, Zheng Gang Zhang

**Affiliations:** ^1^Department of Neurology, Henry Ford Health, Detroit, MI, United States; ^2^Department of Biostatistics and Research Epidemiology, Henry Ford Health, Detroit, MI, United States; ^3^Department of Pathology, Henry Ford Health, Detroit, MI, United States; ^4^Department of Physics, Oakland University, Rochester, MI, United States

**Keywords:** Schwann cells, engineered extracellular vesicles, microRNAs, peripheral neuropathy, diabetes, mice

## Abstract

Extracellular vesicles (EVs) derived from healthy Schwann cells (SC-EVs) ameliorate peripheral neuropathy in diabetic mice and rescue sciatic nerve function in Schwann cell Dicer knockout mice in part via SC-EV cargo miRNAs. Among these miRNAs, miR-214 repairs nerve damage. The present study investigated whether engineered SC-EVs with elevated miR-214 (214-EVs), further amplify the therapeutic effect of naïve SC-EVs (naïve-EVs) on reducing diabetic peripheral neuropathy (DPN) in a mouse model of high-fat diet (HFD)-streptozotocin (STZ) induced type 2 diabetes. Compared to naïve-EVs, 214-EVs significantly improved motor and sensory nerve conduction velocity of the sciatic nerve and thermal latency, which were associated with increased intraepidermal nerve fiber density, axonal diameter, and myelin thickness in the sciatic nerve. Quantitative RT-PCR and Western blot analyses of sciatic nerve tissues showed that, compared to naïve-EVs, 214-EVs significantly increased miR-214 levels and downregulated axonal inhibitory protein PTEN and the myelination inhibitory protein cJUN. Furthermore, 214-EVs markedly suppressed neuroinflammation by decreasing CD68 + macrophages and inactivating the TLR4/NF-κB signaling pathway. Collectively, our findings demonstrate that miR-214-enriched SC-EVs are superior to naïve-EVs to ameliorate DPN and represent a promising EV-based therapeutic strategy.

## Introduction

Diabetic peripheral neuropathy (DPN), one of the most common complications of diabetes, is characterized by progressive nerve damage that primarily affects sensory nerves and eventually leads to motor dysfunction (Feldman et al., 2019; Yang et al., 2025). Currently, there are no effective therapies to cure DPN, highlighting the urgent need for novel and more effective therapeutic strategies.

Extracellular vesicles (EVs) play a crucial role in intercellular communication by delivering their cargo to recipient cells and consequently leading to alteration of recipient cell function (Bang and Thum, 2012; van Niel et al., 2018; Mathieu et al., 2019; Liu and Wang, 2023). EVs derived from healthy Schwann cells (SC-EVs) have been employed in treating peripheral nerve injury and neurodegenerative disease, making them a promising target for regenerative medicine and drug delivery strategies (Lopez-Verrilli et al., 2013; Xia et al., 2020; Ghosh and Pearse, 2023; Zhu S. et al., 2023).

Our previous studies demonstrated that SC-EVs ameliorate DPN in diabetic db/db mice, and the SC-EV cargo including miRNAs contribute to their therapeutic effect (Wang et al., 2020). Furthermore, transgenic mice with the conditional and inducible ablation of the key miRNA biogenesis gene, Dicer, in proteolipid protein (PLP) expressing Schwann cells (PLP-cKO) exhibit a significant reduction of miRNAs and genes involved in myelination and axonal function, resulting in the development of peripheral neuropathy (Wang et al., 2024). However, treatment of PLP-cKO mice with SC-EVs elevates some of the downregulated miRNAs and suppresses myelination and axonal inhibitory genes, leading to recurring peripheral neuropathy. These finding further support the role of SC-EVs cargo miRNAs as key mediators of their therapeutic effects in peripheral neuropathy.

Among miRNAs that mediate the sciatic nerve function, miR-214 plays a crucial role. Diabetic animals exhibit significant downregulation of miR-214 in the sciatic nerve and dorsal root ganglion (DRG) neurons (Pang et al., 2021). Diabetic patients also show miR-214 downregulation in their serum (Xiao et al., 2021; Yan et al., 2024; Ding et al., 2025). Systemic injection of a lentivirus carrying miR-214 has been demonstrated to ameliorate diabetic neuropathy by reducing inflammation and promoting nerve repair in rats (Pang et al., 2021). miR-214 overexpressed in Schwann cell-like cells (SCLC) derived from human amniotic mesenchymal stem cells (MSCs) enhance functional recovery after sciatic nerve injury (Chen et al., 2019). EVs derived from muscle stem cells overexpressing miR-214 facilitate sciatic nerve regeneration following crush injury (Zeng et al., 2023). However, the therapeutic potential of engineered miR-214 enriched SC-EVs for DPN has not been investigated. Using a mouse model of high-fat diet (HFD)-streptozotocin (STZ) induced type 2 diabetes (HFD-STZ-T2D), the present study tested the hypothesis that engineered SC-EVs with elevated miR-214 amplify the therapeutic benefit of naïve SC-EVs for DPN.

## Materials and methods

### Transfection of miR-214 into Schwann cells

Schwann cells (M1700-57, ScienCell) isolated from postnatal day 8 C57BL/6 mouse sciatic nerves were cultured in Schwann cell medium (1701, ScienCell). To overexpress miR-214 in Schwann cells (SCs), a lentiviral vector-carrying the human pre-microRNA expression construct Lenti-miR-214 (hsa-miR-214 ACAGCAGGCACAGACAGGCAGT, PMIRH 214PA-1, System Biosciences) was packaged using Lenti-X™ Packaging Single Shots (631276, Takara Bio USA, Inc.) according to the manufacture’s protocols. A lentiviral vector-carrying scramble construct (PMIRH000PA-1, System Biosciences) was used as a control. To maintain the purity of transfected Schwann cells, cells were cultured in the presence of 1.1 ug/ml puromycin. When Schwann cells reached approximately 60%∼80% confluence, culture medium was changed to a medium containing 5% exosome-depleted fetal bovine serum-contained medium (SBI System Bioscience), and Schwann cells were maintained for an additional 48 h. The conditioned medium was then collected for isolation of EVs.

### Generation and characterization of SC-EVs

Extracellular vesicles (EVs) were isolated using a differential ultracentrifugation approach and characterized in compliance with the guideline of Minimal information for studies of extracellular vesicles 2023 (Welsh et al., 2024). Briefly, the collected supernatant was filtered through a 0.22 μm filter before being centrifuged at 10,000 *g* for 30 min. Subsequently, ultracentrifugation was conducted at 100,000 g (Optima XE-100 Ultracentrifuge, SW 32 Ti Rotor) for 2 h, and the resulting pellet was resuspended with 100 μl of sterilized saline. The concentration and size of EVs were determined using the NanoSight analysis system (Malvern Panalytical). Western blot analysis was performed to measure proteins of Alix, heat shock protein (HSP 70), TSG101, CD9, CD63, and calnexin in the isolated EVs (Wang et al., 2020; Zhang et al., 2025).

Transmission electron microscopy (TEM) was performed to examine the ultrastructure of the isolated EVs (Zhang et al., 2025). Briefly, freshly isolated EVs were placed onto copper grids coated with Formvar carbon film (Catalog #FCF400-Cu, Electron Microscopy Sciences, Hatfield, PA, USA) and allowed to adhere for 45 s. Unbound particles were removed by rinsing the grids twice with distilled water. The samples were then briefly stained with 1% osmium tetroxide (OsO4, Catalog #19100, Electron Microscopy Sciences) for 30 s. Prepared grids were subsequently examined under a JEOL 1400 Flash transmission electron microscope (JEOL, Tokyo, Japan).

### Animals

All experimental procedures were approved by the Institutional Animal Care and Use Committee of Henry Ford Hospital (IACUC #1502) and were conducted according to NIH Guidelines for the Care and Use of Laboratory Animals. All experiments and data analyses were conducted by investigators who were unaware of the treatment assignments.

To induce the T2D model, male C57BL/6 mice at the age of 10 weeks were fed a 60-kcal% high-fat diet (HFD, D12492; Research Diets). After 8 weeks on HFD, mice were treated with two low doses of streptozotocin (STZ, 75 mg/kg followed with 50 mg/kg 3 days later) (Yorek et al., 2015, 2017). Mice with blood glucose >250 mg/dl were considered diabetic. These mice remained on the HFD for the duration of the study. WT group was fed the standard diet for the duration of the study. To evaluate the effect of naïve-EVs, 214-EVs, and Scra-EVs on neurological recovery of DPN mice, 16 weeks post HFD/STZ treatment, the mice were randomly divided into one of the following treatment groups: (1) WT + saline (WT); (2) T2D + saline (T2D); (3) T2D + naïve-SC-EVs (EVs); (4) T2D + miR-214 enriched SC-EVs (214-EVs); (5) T2D + scramble-SC-EVs (Scra-EVs), where *n* = 10 mice/group was selected to ensure the study had an 80% power to detect an effect size of 1.48, assuming two-sided test, alpha = 0.05 and an equal-space means (Cohen, 1988). EVs (2 × 10^10^particles in 0.2 ml saline/mouse) or an equal volume of saline were intravenously administered via a tail vein once every week for 8 consecutive weeks, respectively. All mice were sacrificed 8 weeks after the initial treatment. The doses of EVs were selected based on our published studies (Wang et al., 2020, 2024).

### Electrophysiological assessments

Motor nerve conduction velocity (MCV) and sensory nerve conduction velocity (SCV) in sciatic nerve were measured every 4 weeks, as previously described (Wang et al., 2012, 2024). Briefly, mice were anesthetized with 1.5% isoflurane, and electrodes were positioned at the sciatic notch and knee. Single square wave current pulses were applied using a pulse stimulator, while simultaneous electromyography recordings were obtained through two sterilized electrodes inserted into the dorsum of the foot. Data were collected using a 4-channel amplifier (Natus UltraPro S100 EMG/NCS/EP Neurodiagnostic System). To maintain a stable body temperature of 37°C ± 1.0°C during the measurements, a water-based heater was used. MCV and SCV were analyzed using the Natus Elite software supplied provided by the manufacturer (Wang et al., 2012, 2024).

### Thermal sensitivity assessment

Thermal sensitivity was evaluated biweekly using the Hargreaves method with a thermal stimulation meter (plantar test, Model 336 TG, IITC Life Science), following established protocols (Wang et al., 2012, 2020, 2024). Mice were allowed to acclimate on a transparent glass surface for at least 20 min before testing. A thermal stimulator was positioned under the plantar surface of the hind paw, and withdrawal latency in response to radiant heat at 15% intensity was measured. Each mouse underwent three trials, with a 15-min interval between measurements. The average withdrawal latency was then calculated for each animal.

## Biochemical analyses

Blood glucose levels were assessed using an instant check meter (Roche Diagnostics).

### Immunohistochemistry and image quantification

For immunofluorescent staining, sciatic nerve tissues were fixed in 4% paraformaldehyde, embedded in paraffin, and sectioned at a thickness of 6 μm. A total of three cross-sections with each at 60 μm interval per animal were employed for immunochemistry study with a primary antibody against CD68 (1:30 dilution, BIO-RAD, Catalog #MCA341) (Wang et al., 2024).

To assess intraepidermal nerve fiber density (IENFs), hind paw plantar skin tissues were fixed in Zamboni Fixative, following our published protocol (Wang et al., 2020; Zhang et al., 2021a). 20 μm-thick cryosections of footpad tissue were immunostained with an anti-protein gene product 9.5 (PGP9.5) antibody (1:1,000; MILLIPORE) and imaged using a FluoView FV 1200 laser scanning confocal microscope (Olympus) with a 40x objective. Images were analyzed using the MicroComputer Imaging Device (MCID) system (Imaging Research Inc.). Nerve fibers crossing the dermal-epidermal junction were counted, and intraepidermal nerve fiber density was expressed as the number of fibers per millimeter of epidermal length (Fan et al., 2020; Wang et al., 2020; Zhang et al., 2021a).

For morphometric analysis of the sciatic nerve, tissue samples were processed, as described previously (Wang et al., 2011, 2020, 2024). Transverse sciatic nerve sections (2 μm thick) were stained with toluidine blue and randomly imaged using a 100 × oil immersion lens (Olympus Optical Co., Ltd.). Myelin sheath thickness, myelinated fiber and axon diameter were quantified using the MCID system, following established protocols (Wang et al., 2011, 2020, 2024).

## *In vitro* experimental protocols

### Assessment of DRG neurite outgrowth

Dorsal root ganglia (DRG) neurons were harvested from 26-week-old WT and T2D mice and cultured under normal glucose (RG, 5 mM) and high glucose (HG, 30 mM) conditions, according to published protocols (Wang et al., 2020, 2024). To evaluate the impact of EVs on neurite outgrowth, DRG neurons (2,000 cells/cm^2^) were plated on glass coverslips and treated with one of the following conditions: (1) WT + saline (WT); (2) T2D + saline (T2D); (3) T2D + naïve-SC-EVs (EVs); (4) T2D + miR-214 enriched SC-EVs (214-EVs); (5) T2D + scramble-SC-EVs (Scra-EVs). EVs were applied at a concentration of 6 × 10^9^ particles/ml. After 72 h in culture, neurons were immunostained with an anti-neurofilament heavy subunit (NF-H) antibody (1:500, BioLegend). Images were captured at 10 × magnification using a digital camera. Neurite outgrowth was quantified by measuring the total neurite length of 20 neurons per group using the MCID system, and the average neurite length was calculated (Wang et al., 2020).

### Assessment of Schwann cell migration

Schwann cells were grown to 90% confluence in six-well plates under normal (5 mM) and high glucose (30 mM) medium. A scratch wound was created using a 10 μL pipette tip, and cells were incubated for 18 h. The wound closure was then assessed by capturing images and measuring the gap distance using the MCID system (Wang et al., 2020).

### Quantitative real-time RT-PCR (qRT-PCR) analysis

Total RNA was extracted from EVs and sciatic nerve tissues using the miRNeasy Mini Kit (Qiagen) and subsequently reverse transcribed. Quantitative real-time RT-PCR (qRT-PCR) was carried out using a TaqMan miRNA assay kit and TaqMan PCR reagents, following the protocol outlined in previous studies (Wang et al., 2020, 2024). The following hydrolysis miRNA primers were used: has-miR-214-3p (Assay ID: 002306, mature sequence: ACAGCAGGCACAGACAGGCAGU). U6 snRNA (Assay ID: 001973, mature sequence: GTGCTCGCTTCGGCAGCACATATACTAAAATTGGAACGATA CAGAGAAGATTAGCATGGCCCCTGCGCAAGGATGACACGC AAATTCGTGAAGCGTTCCATATTTT). The relative expression levels of miRNAs were determined using the 2-ΔΔCt method (Livak and Schmittgen, 2001), with U6 snRNA (Applied Biosystems) serving as the endogenous control.

### Western blot analysis

Western blots were conducted following established protocols (Wang et al., 2015, 2020). In brief, samples were lysed and the protein concentration in the supernatant was quantified using a bicinchoninic acid (BCA) assay kit (Pierce Biotechnology). Equal amounts of proteins were separated by SDS-PAGE and transferred onto Polyvinylidene difluoride (PVDF) membranes. After blocking, the membranes were incubated overnight with primary antibodies at 4°C and followed with secondary antibody (1:1000). A complete list of antibodies employed in this study is provided in Supplementary Table 1. The signals were detected using an enhanced chemiluminescence detection kit (Pierce Biotechnology) and visualized with the FluorChem E System (ProteinSimple).

### Statistical analysis

Generalized estimating equations (GEE) were used to evaluate the effects of group and time on neurological test outcomes. The interaction between group and time was assessed first. If the interaction was not significant, the main effects of group and time were analyzed separately. For multiple group comparisons, one-way analysis of variance (ANOVA) followed by Tukey’s multiple comparisons test was performed. For pairwise group comparisons, Student’s *t*-tests were used. Data are presented as mean ± standard error of the mean (SE). A *p*-value of < 0.05 was considered statistically significant.

## Results

### Engineered miR-214 enriched SC-EVs (214-EVs) are superior to naïve-SC-EVs to ameliorate DPN

**FIGURE 1:**
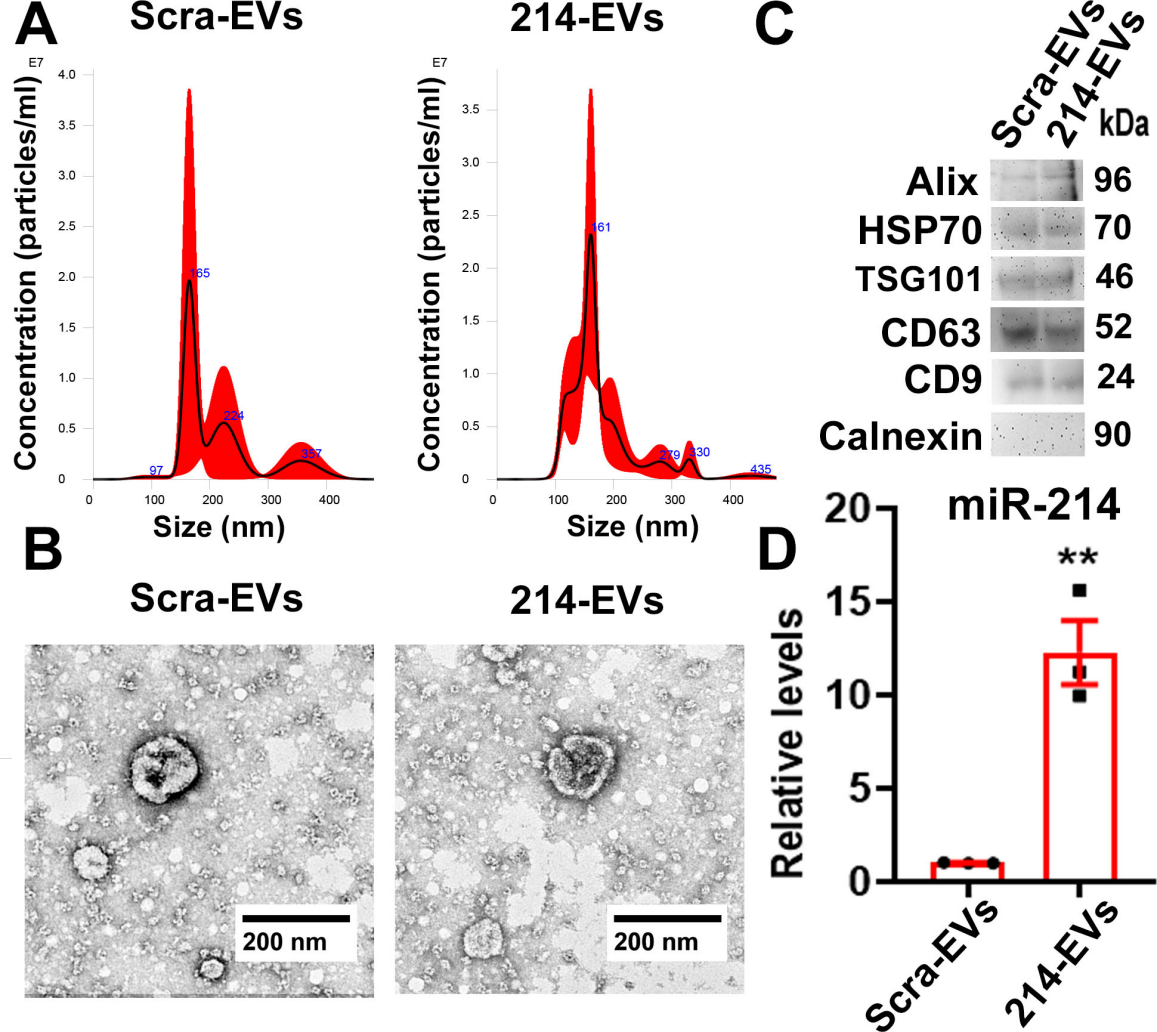
Characterization of engineered SC-EVs enriched with miR-214. Panel **(A)** shows representative nanoparticle tracking analysis (NTA) of the size distribution of EVs isolated from the conditioned medium of SCs transfected with a scramble vector (Scra-EVs) and a miR-214 overexpression vector (214-EVs). Panel **(B)** shows representative TEM images of the EV morphology of Scra-EVs and 214-EVs. Panel **(C,D)** show Western blot analysis **(C)** and quantitative RT-PCR data **(D),** illustrating the expression of EV marker proteins and miR-214 levels in Scra-EVs and 214-EVs, respectively. Data are expressed as the mean ± SE. **p < 0.01 vs. Scra-EVs. *n* = 3/group.

To investigate the effect of 214-EVs on DPN, we generated and characterized 214-EVs. EVs were isolated from exosome free conditional media of cultured Schwann cells transfected with a miR-214-containing lentiviral vector or a control scramble vector by means of differential ultracentrifugation (Wang et al., 2020, 2024). Following the EV guidelines (MISEV2023) (Welsh et al., 2024), the isolated EVs were characterized using Nanosight nanoparticle tracking analysis (NTA), TEM, and Western blotting. We found that there were no significant differences in size distributions and morphology between 214-EVs and EVs isolated from the media of Schwann cells transfected with the control scramble vector (Scra-EVs). These EVs contained EV marker proteins (Alix, HSP70, TSG101, CD9 and CD63), but not Calnexin, a negative EV control protein (Figure 1). Quantitative RT-PCR analysis revealed that miR-214 levels were significantly elevated in 214-EVs compared to Scra-EVs (Figure 1), indicating that we successfully generated SC-EVs enriched with miR-214.

**FIGURE 2:**
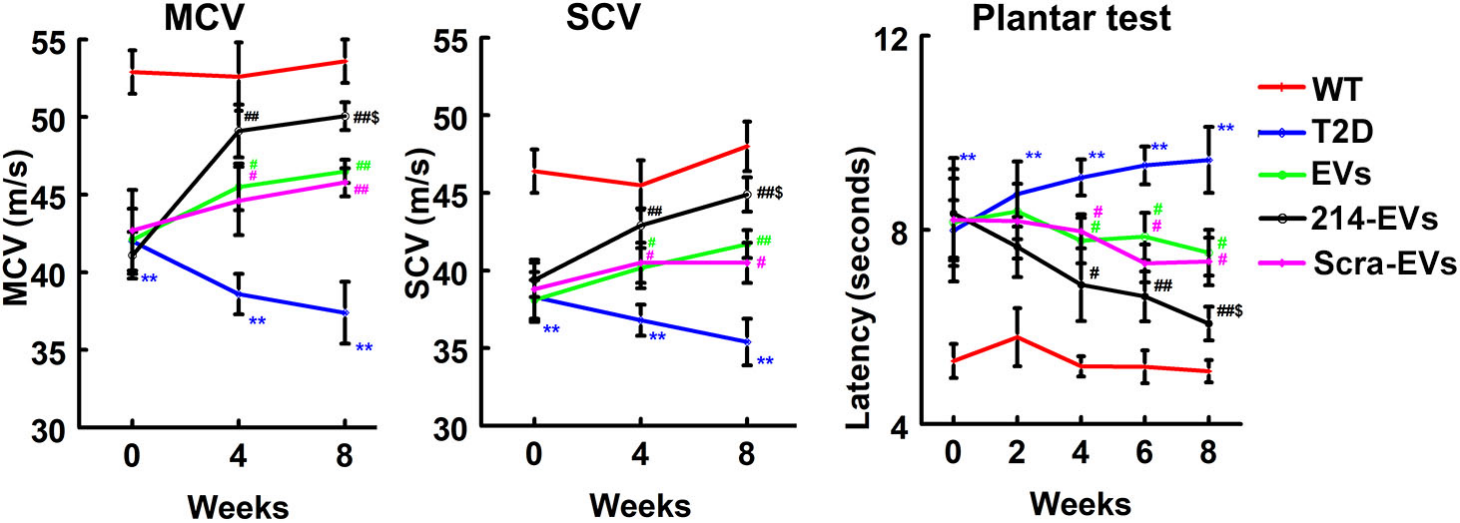
The therapeutic effect of miR-214 enriched SC-EVs on DPN is superior to Scra-EVs. Treatment of T2D mice with naïve-SC-EVs (EVs, green) and scramble-SC-EVs (Scra-EVs, purple) improve neurological function measured by MCV, SCV, and Plantar test, respectively. miR-214 enriched SC-EVs (214-EVs, black) treatment induces a more robust improvement of neurological function in peripheral nerve than treatment with Scra-EVs. **p < 0.01 versus the wild type mice treated with saline (WT, red) and #*p* < 0.05, ##*p* < 0.01 versus the diabetic mice treated with saline (T2D, blue), $*p* < 0.05 versus the T2D mice treated with Scra-EVs. *n* = 10 mice/group.

Next, we examined the effect of 214-EVs on DPN of the HFD/STZ-induced T2D mice. Compared to wild-type mice (WT), 16 weeks post-HFD/STZ administration, the HFD/STZ-induced T2D mice displayed significant DPN symptoms, characterized by marked reductions in motor and sensory nerve conduction velocities (MCV and SCV), and thermal sensitivity as measured by plantar test (Figure 2). We thus randomly treated (I.V.) HFD/STZ mice with 214-EVs and Scra-EVs weekly for 8 consecutive weeks starting 16 weeks post HFD/STZ when mice exhibited DPN. Compared to the saline treatment, HFD/STZ mice treated with naïve-SC-EVs and Scra-EVs exhibited significant attenuation of diabetes-induced reductions in MCV and SCV, along with improvements in thermal sensitivity starting 4 weeks after the treatment, and the improvement persisted for at least 8 weeks. These data are consistent with our previous findings of the therapeutic efficacy of SC-EVs in DPN animal models. However, 214-EVs treatment led to further significant improvement of neurological function at 8-weeks post-treatment compared to Scra-EVs (Figure 2). Additionally, treatment with 214-EVs and Scra-EVs, did not significantly affect blood glucose levels and animal body weight (Table 1). These data indicate that the 214-EVs’ therapeutic benefit is superior to naïve-SC-EVs.

**TABLE 1:** Effect of EVs on body weight and blood glucose.

Time	WT	T2D	EVs	214-EVs	Scra-EVs
**Body weight (g)**					
0W	30.8 ± 0.6	43.1 ± 2.2*	45.6 ± 1.2*	44.0 ± 2.7*	44.6 ± 1.6*
4W	30.9 ± 0.8	48.2 ± 2.9*	48.9 ± 0.7*	46.3 ± 2.1*	46.0 ± 1.7*
8W	31.9 ± 0.6	49.3 ± 3.3*	49.9 ± 0.5*	47.9 ± 2.6*	47.8 ± 2.5*
**Blood glucose (mg/dl)**					
0W	136.4 ± 6.5	253.2 ± 19.6*	264.5 ± 15.8*	264.2 ± 28.3*	271.3 ± 29.6*
4W	140.8 ± 8.24	283.6 ± 42.8*	281.6 ± 18.1*	273.8 ± 39.6*	270.3 ± 13.6*
8W	132.5 ± 7.8	311.1 ± 40.9*	307.8 ± 15.6*	302.6 ± 23.1*	311.6 ± 37.2*

*p < 0.01 versus the wild type mice treated with saline (WT). *n* = 10 mice/group. T2D, diabetic mice treated with saline; EVs, diabetic mice treated with naïve-SC-EVs; 214-EVs, diabetic mice treated with miR-214 enriched SC-EVs; Scra-EVs, diabetic mice treated with scramble-SC-EVs; W, week.

### Engineered miR-214 enriched SC-EVs (214-EVs) amplify the effect of naïve-SC-EVs on reduction of diabetic-induced sciatic nerve damage

**FIGURE 3:**
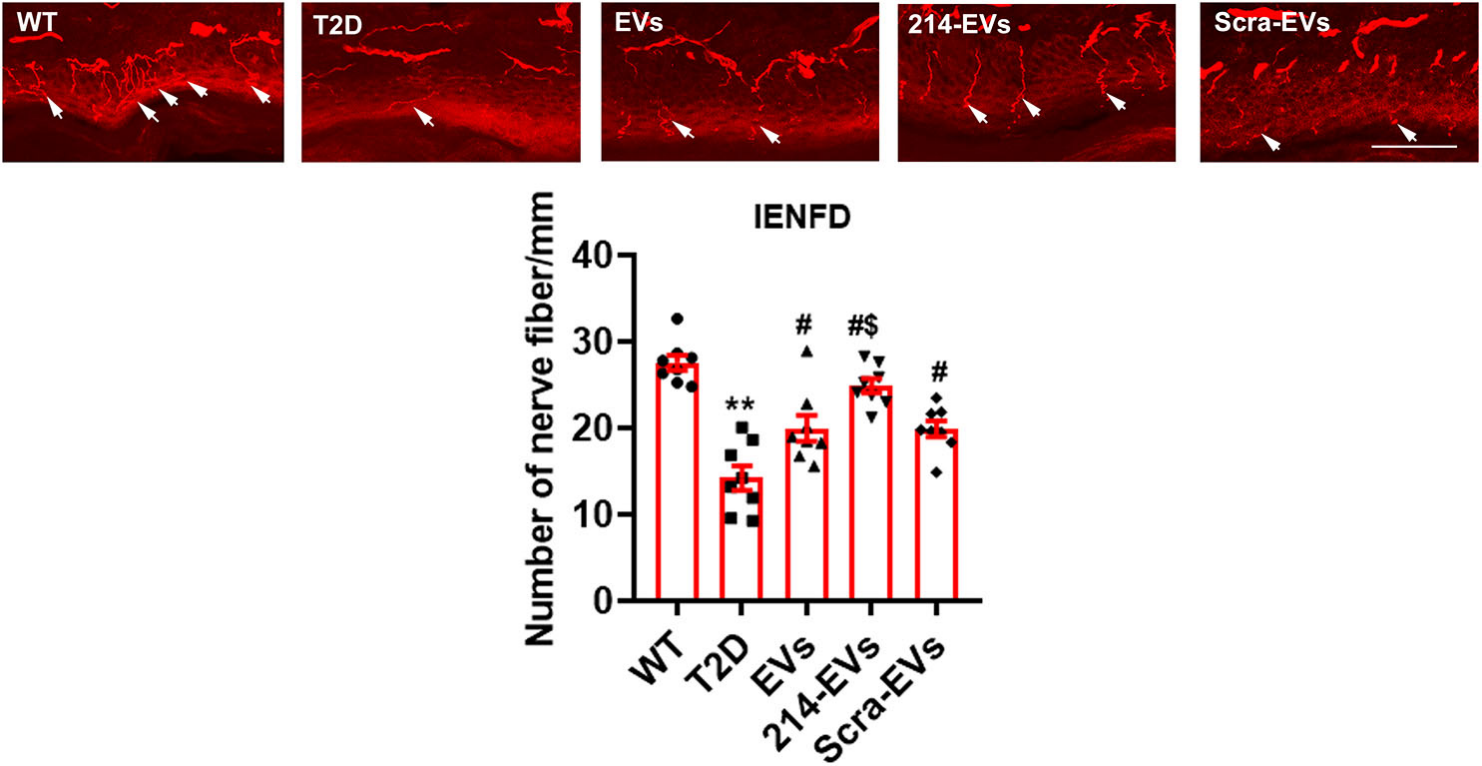
miR-214 enriched SC-EVs treatment increases intraepidermal nerve fiber density (IENFD). Representative images show PGP immunoreactive intraepidermal nerve fibers (red, arrows) in hind paw plantar skin from wild-type mice (WT), diabetic mice treated with saline (T2D), diabetic mice treated with naïve-SC-EVs (EVs), diabetic mice treated with miR-214 enriched SC-EVs (214-EVs) and diabetic mice treated with scramble-SC-EVs (Scra-EVs). Histogram represents the quantitative data of the nerve fiber density under various conditions. Bar = 50 μm. *n* = 8 mice/group. **p < 0.01 versus wild type mice treated with saline (WT). #*p* < 0.05 versus diabetic mice treated with saline (T2D). $*p* < 0.05 versus the T2D mice treated with Scra-EVs.

To examine whether 214-EVs treatment affects nerve fibers, morphometric changes of nerve fibers were analyzed. Immunohistochemical analysis revealed that compared to WT mice, HFD/STZ mice at age of 34 weeks exhibited a significant reduction in intraepidermal nerve fiber density (IENFD), as quantified by PGP9.5-positive sensory nerve fibers in footpad tissues. Naïve-SC-EVs and Scra-EVs treatment significantly augmented IENFD compared to saline treatment. However, treatment with 214-EVs led to a further significant increase in IENFD compared to the Scra-EVs treatment, indicating enhanced distal nerve fiber regeneration (Figure 3).

**FIGURE 4:**
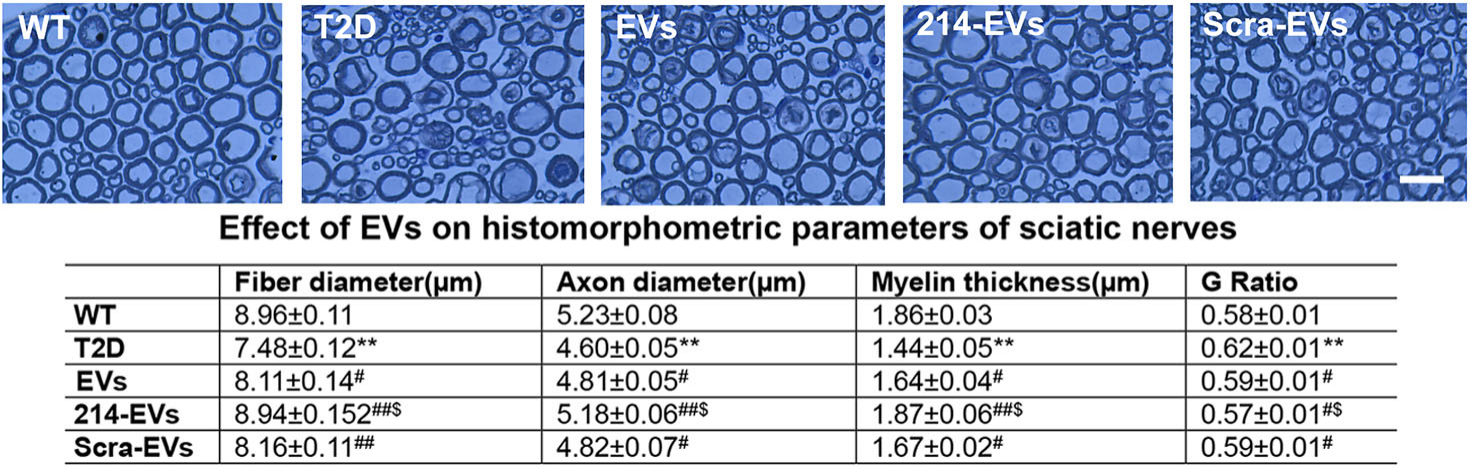
miR-214 enriched SC-EVs treatment amplifies the impact of SC-EVs on histomorphometric changes of sciatic nerves. Representative images of semi-thin toluidine blue-stained cross sections of sciatic nerves from wild-type (WT), diabetic mice treated with saline (T2D), diabetic mice treated with naïve-SC-EVs (EVs), diabetic mice treated with miR-214 enriched SC-EVs (214-EVs) and diabetic mice treated with scramble-SC-EVs (Scra-EVs). Table shows the quantitative data of histomorphometric parameter of toluidine blue-stained of sciatic nerve. Bar in *E* = 25 μm, *n* = 8/group. **p < 0.01 versus the wild type mice treated with saline (WT) and #*p* < 0.05, ##*p* < 0.01 versus the diabetic mice treated with saline (T2D), $*p* < 0.05 versus the T2D mice treated with Scar-EVs.

Morphometric analysis of sciatic nerves using toluidine blue staining revealed that HFD/STZ mice exhibited substantial reduction of nerve fiber diameter and myelin sheath thickness, and elevated g-ratio (axon diameter/fiber diameter). Treatment with naïve-SC-EVs and Scra-EVs significantly improved these parameters compared to saline treated HFD/STZ mice. Importantly, 214-EVs treatment resulted in further significant improvements in nerve fiber diameter, myelin thickness, and g-ration, suggesting superior promotion of axonal regeneration and remyelination in diabetic sciatic nerve tissues (Figure 4).

### Engineered miR-214 enriched SC-EVs (214-EVs) amplify the effect of naïve-SC-EVs on Schwann cell migration and DRG neurite outgrowth *in vitro*

**FIGURE 5:**
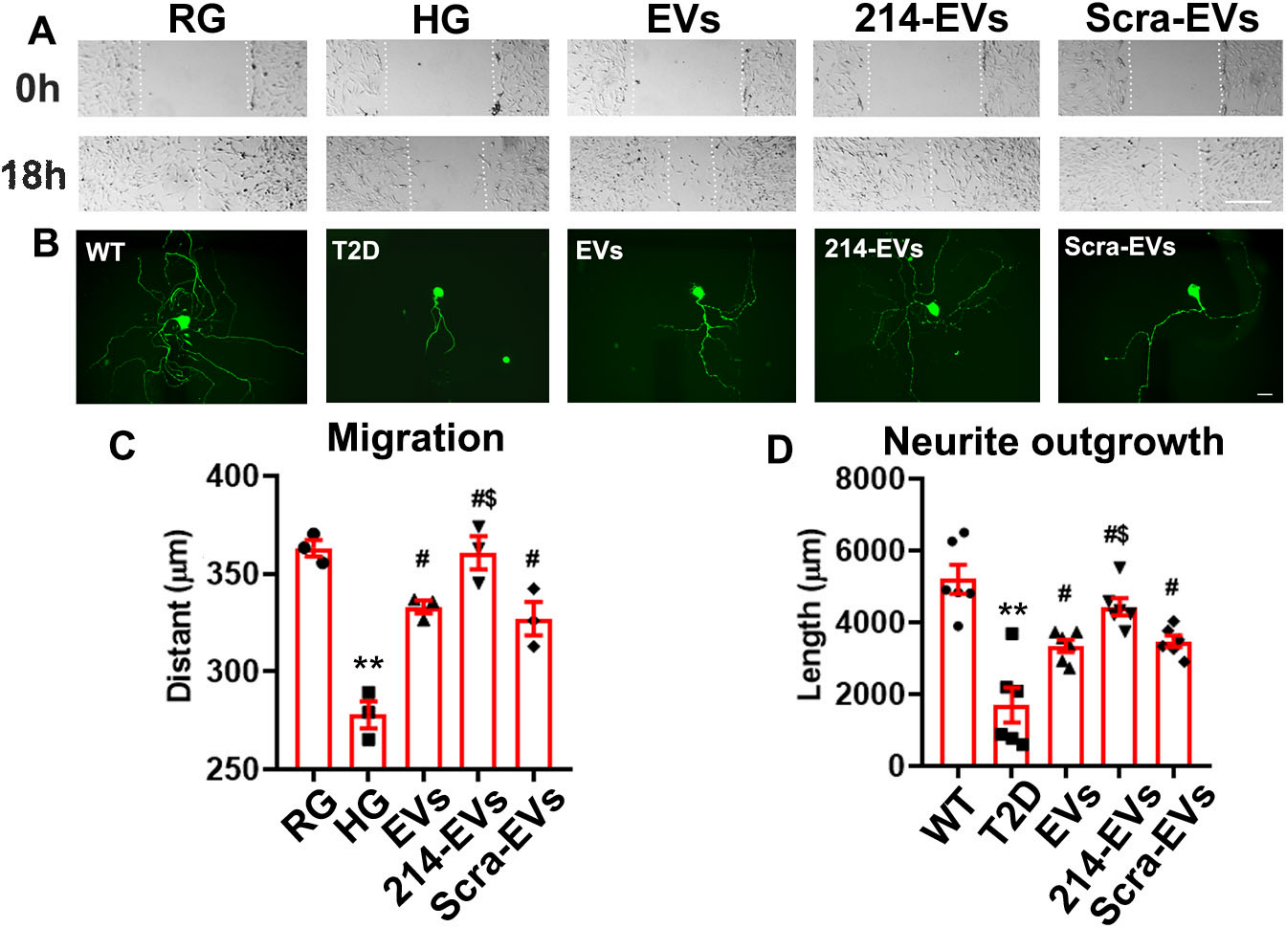
Effect of miR-214 enriched SC-EVs on Schwann cell migration and DRG neurite outgrowth *in vitro*. Panel **(A)** shows representative images of migration of Schwann cells treated with regular glucose (RG), high glucose (HG) with naïve-SC-EVs (EVs), miR-214 enriched SC-EVs (214-EVs) or scramble-SC-EVs (Scra-EVs). Panel **(B)** shows representative images of neurofilament H (NFH) + neurite of DRG neurons derived from wild-type mice (WT), diabetic mice (T2D), diabetic mice treated with naïve-SC-EVs (EVs) or scramble-SC-EVs (Scra-EVs). Panel **(C,D)** show quantitative data of migration of Schwann cells (**C**, *n* = 3) and neurite length of DRG neuron (**D**, *n* = 6) under different culture conditions. Bar in *A* = 100 μm, Bar in B = 50 μm. **p < 0.01 versus the regular glucose (RG) or WT mice (WT). #*p* < 0.05 versus high glucose (HG) group or T2D mice, respectively. $*p* < 0.05 versus high glucose (HG) or T2D mice treated with Scar-EVs.

To investigate the cellular effects of 214-EVs on Schwann cells and DRG neurons, we conducted *in vitro* experiments. DRG neurons isolated from 26-week-old T2D mice and Schwann cells were incubated with high glucose (30 mM) with or without SC-EVs (6 × 10^9^ particles/ml). We found that naïve-SC-EVs and Scra-EVs significantly decreased the inhibitory effects of high glucose on Schwann cell migration and DRG neurite outgrowth. However, treatment with 214-EVs resulted in substantially enhanced Schwann cell migration and DRG neurite outgrowth compared to Scra-EVs treated cells (Figure 5). These *in vitro* results support for our *in vivo* finding, indicating that 214-EVs amplify the effect of naïve-SC-EVs on Schwann cell migration and DRG neurite outgrowth.

### Engineered miR-214 enriched SC-EVs (214-EVs) increase miR-214 levels and suppress axonal and myelination inhibitory proteins in the sciatic nerve tissues

**FIGURE 6:**
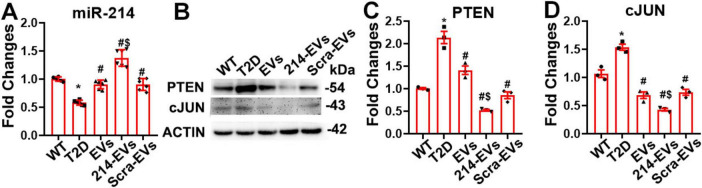
Cargo miR-214 mediates the effect of miR-214 enriched SC-EVs on suppressing axonal inhibitor protein PTEN and myelin inhibitor protein cJUN in sciatic nerve tissue. Panel **(A)** shows quantitative RT-PCR data of the levels of miR-214 in sciatic nerve tissues (*n* = 6/group). Panel **(B,C)** show representative images of Western blot analysis (**B**) and relative expression levels of PTEN **(C)** and cJUN (**D**) in the sciatic nerve tissue. *n* = 3 mice/group. *p < 0.05 versus wild-type mice treated with saline (WT). #*p* < 0.05 versus diabetic mice treated with saline (T2D). $*p* < 0.05 versus the T2D mice treated with scramble-SC-EVs (Scra-EVs). EVs, diabetic mice treated with naïve-SC-EVs; 214-EVs, diabetic mice treated with miR-214 enriched SC-EVs.

Quantitative RT-PCR analysis of sciatic nerve tissues revealed that miR-214 expression was substantially reduced in HFD/STZ mice compared to WT controls. Compared with saline treatment, naïve-SC-EVs and Scra-EVs significantly increased miR-214 levels. However, 214-EVs treatment led to a further significant increase in miR-214 expression in HFD/STZ mice (Figure 6).

PTEN and c-Jun have been identified as target genes of miR-214, known to inhibit axonal growth and myelination, respectively (Chen et al., 2019; Zeng et al., 2023). Western blot analysis of sciatic nerve tissues showed markedly elevated PTEN and c-Jun protein levels in HFD/STZ mice compared to WT controls. Treatment with naïve-SC-EVs and Scra-EVs significantly reduced the expression of both inhibitory proteins, which is consistent with our previous studies (Wang et al., 2020, 2024). However, treatment with 214-EVs resulted in a further reduction in PTEN and c-Jun levels (Figure 6). These data demonstrate that diabetes suppresses miR-214 expression and elevates its target proteins, PTEN and cJUN, in sciatic nerve tissue. The enhanced therapeutic effect of 214-EVs appears to be mediated through increased delivery of miR-214, which more effectively downregulates PTEN and c-Jun, thereby promoting axonal regeneration and remyelination.

### Engineered miR-214 enriched SC-EVs (214-EVs) amplify the effect of naïve-SC-EVs on attenuate inflammatory response in DPN

**FIGURE 7:**
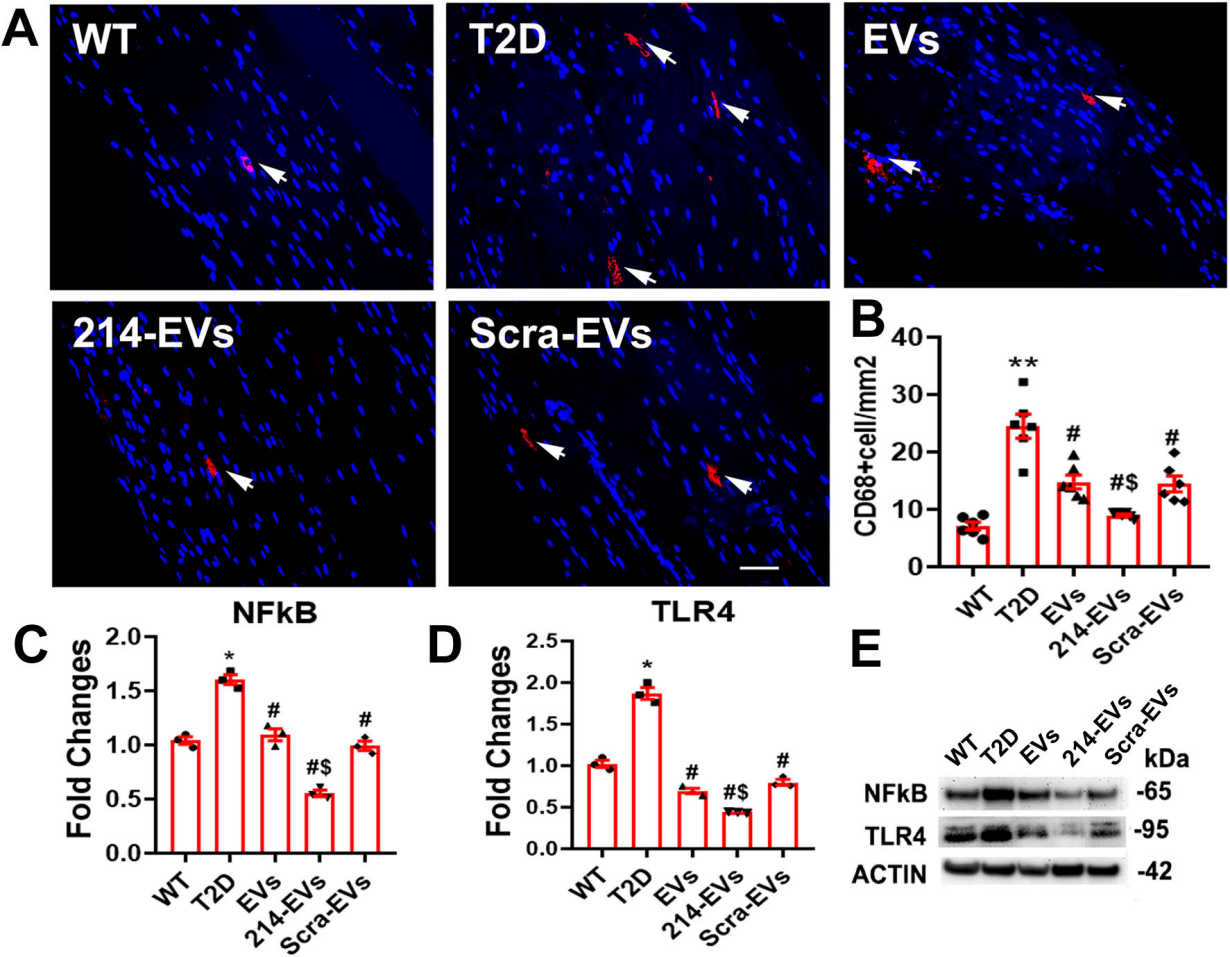
Effect of miR-214 enriched SC-EVs treatment on inflammatory response. Panel **(A,B)** show representative images and quantitative data of active CD68 + macrophages (CD68 + cells) in the sciatic nerve. *n* = 6 mice/group, Bar = **2**5 μm. Panel **(C–E)** show representative images of Western blot analysis (**E**) and relative expression levels of NFkB **(C)** and TLR4 **(D)** in sciatic nerve. *n* = 3/group. *p < 0.05, **p < 0.01 versus the wild type mice treated with saline (WT) and #*p* < 0.05 versus the diabetic mice treated with saline (T2D), $*p* < 0.05 versus the T2D mice treated with scramble-SC-EVs (Scra-EVs). EVs, diabetic mice treated with naïve-SC-EVs; 214-EVs, diabetic mice treated with miR-214 enriched SC-EVs.

Inflammation plays a crucial role in the pathogenesis of DPN, with macrophages acting as key neuroinflammatory regulators affecting peripheral nerve tissue integrity (Farmer et al., 2012; Panou et al., 2025). Immunohistochemical analysis revealed a significant increase in CD68 + macrophage accumulation in the sciatic nerves of HFD/STZ mice. Treatment with naïve-SC-EVs and Scra-EVs significantly reduced CD68 + cell infiltration, however, 214-EVs showed a superior reduction in CD68 + cells compared to Scra-EVs treatment (Figure 7).

Western blot analysis of sciatic nerve tissue showed that HFD/STZ mice exhibited elevated expression of pro-inflammatory mediators, TLR4 and NFκB. Naïve-SC-EVs and Scra-EVs treatment reduced these inflammatory markers. However, 214-EVs completely abolished the diabetes-induced elevation of TLR4 and NFκB expression (Figure 7). Bioinformatic analysis identified TLR4 as a predicted target of miR-214, suggesting that 214-EVs suppress neuroinflammation by decreasing CD68 + macrophages and inactivating the TLR4/NF-kB signaling pathway.

## Discussion

In this study, we demonstrated that engineered miR-214 enriched SC-EVs significantly amplify the therapeutic efficacy of naïve-SC-EVs on DPN in a mouse model of HFD/STZ induced T2D. The improved neurological function was associated with reduced sciatic nerve damage and increased intraepidermal nerve fiber density. Downregulation of inhibitors of axonal growth and myelination and inactivation of the TLR4/NF-κB signaling pathway mediated neuroinflammation could underscore the 214-EV therapeutic effect on DPN.

In our previous studies, we demonstrated that SC-EV cargo miRNAs contribute to the therapeutic effect on DPN in diabetic db/db mice and on peripheral neuropathy in Schwann cell Dicer-knockout mice (Wang et al., 2020, 2024). miR-214 is a key modulator of peripheral nerve function (Pang et al., 2021; Zeng et al., 2023). Preclinical studies and patient data show that miR-214 deficiency is highly associated with DPN (Ding et al., 2025). For example, a significant downregulation of miR-214 is detected in the sciatic nerve and DRG neuron of diabetic animals (Pang et al., 2021) and diabetic patients exhibit substantial reduction of serum levels of miR-214 (Qi et al., 2020; Xiao et al., 2021; Ding et al., 2025). Additionally, systemic administration of a lentiviral vector encoding miR-214 attenuates neuropathy in diabetic rats by reducing inflammation and promoting nerve repair (Pang et al., 2021). Others also reported that miR-214 overexpression in SCLCs derived from human amniotic MSC enhances functional recovery following sciatic nerve injury (Chen et al., 2019). Using a set of experiments, the present study provides strong evidence showing that the therapeutic benefit of engineered SC-EVs carrying enriched 214 on DPN is superior to naïve-SC-EVs.

Compared to synthetic miRNA mimics or viral vectors, engineered EVs provide a more stable, precise, and efficient approach for miRNA delivery, leading to superior therapeutic outcomes (Luan et al., 2017; Sun et al., 2023). In the present study, we developed engineered SC-EVs carrying enriched miR-214, which did not alter the fundamental characteristics of the SC-EVs, such as size distribution, morphology, and specific markers. However, treatment with 214-EVs resulted in further improvement in nerve conduction velocity and thermal sensitivity compared to naïve-SC-EVs. These functional benefits were associated with increased epidermal nerve fiber density and improved axonal integrity and remyelination in sciatic nerve tissues. Supporting our *in vivo* finding, *in vitro* assays showed that 214-EVs further promoted SC migration and DRG neurite outgrowth.

As outlined in the MISEV 2018 and 2023 guidelines, currently available methods for isolating EVs cannot ensure complete purity or subtype specificity within the EV population. In the present study, we conducted NTA, TEM and immunodetection of established EV markers to assess the size, morphology, and identity of the isolated vesicles. These complementary approaches indicate that the isolated vesicles are enriched in EVs. However, due to the limitations of current isolation techniques, we cannot rule out the potential roles of non-exosome vesicles in our findings.

Our results are consistent with growing evidence that engineered EVs enriched with specific miRNAs offer enhanced therapeutic efficacy in models of neurological diseases (Xin et al., 2017; Fan et al., 2021; Zeng et al., 2023; Zhang et al., 2025). For example, miR-17-92 cluster-enriched MSC-EVs employed for preclinical treatment stroke and traumatic brain injury (TBI) have demonstrated that miRNA cargo significantly enhances neurovascular plasticity and functional recovery (Xin et al., 2017; Zhang et al., 2021b). Zeng et al. reported that EVs derived from muscle stem cells overexpressing miR-214 enhance sciatic nerve regeneration following crush injury (Zeng et al., 2023). Moreover, treatment of DPN with MSC-EVs enriched with miR-146a provide amplified therapeutic benefit (Fan et al., 2021).

The biological effects of EVs on recipient cells involves multiple steps, including the uptake of EVs and intracellular transport of their cargo. Cargo miRNA can effectively downregulate target genes in the recipient cells (Kwok et al., 2021). Our published studies demonstrated that systemic administration of SC-EVs are taken up by the sciatic nerves and deliver their miRNA cargo including miR-21, -27a, -138 and -146a, that target genes, leading to improvements of peripheral neuropathy (Wang et al., 2020, 2024). The current study shows that 214-EVs significantly elevated miR-214 levels in sciatic nerves tissue, which was associated with suppression of its target proteins, PTEN and c-Jun (Chen et al., 2019; Zeng et al., 2023). These proteins are well-established negative regulators of nerve regeneration, PTEN inhibits axonal growth, while c-JUN is associated with demyelination (Parkinson et al., 2008; Ohtake et al., 2015). Inhibition of PTEN promotes axonal regrowth (Ohtake et al., 2015; Zhou et al., 2020), and downregulation of c-JUN facilitates remyelination in peripheral nerve injury (Parkinson et al., 2008; Chen et al., 2019).

In addition to the effect of miR-214 on neuroprotection, miR-214 exerts anti-inflammatory effects in various diseases (Pang et al., 2021; Xia et al., 2024). Recently work by Xia et al reported that miR-214 ameliorates neuroinflammation after spinal cord injury by targeting the Nmb/Cav3.2 pathway (Xia et al., 2024). Furthermore, Lan et al showed that EV-derived from chondrocytes overexpressing miR-214 facilitates M2 macrophage polarization via ATF4/TLR4 axis (Lan et al., 2023). Consistent with these findings, our data demonstrate that 214-EVs more effectively reduce neuroinflammation in STZ/HFD mice than naïve-SC-EVs by suppressing CD68 + macrophage infiltration and inactivating the pro-inflammatory TLR4/NFkB signaling pathway, suggesting that in addition to acting on axons and myelination, 214-EVs reduce inflammation in DPN.

The present study has multiple limitations. DPN is associated with endothelial dysfunction and impaired blood flow. miR-214 has been shown to promote angiogenesis and improve endothelial cell function, potentially enhancing nerve perfusion (van Balkom et al., 2013; Zhu W. et al., 2023). Whether the changes in the neurovascular remodeling contribute to functional recovery following 214-EVs treatment warrants further studies.

The miR-214-3p forms a cluster with the miR-199a-5p cluster, thus overexpression of miR-214 could affect miR-199a that has been shown to target TLR4. Additionally, overexpression of miR-214 could potentially affect other cargo miRNAs and proteins. Future studies will include unbiased miRNA and proteomic profiling to compare engineered 214-EV and naïve EV cargo for dissecting relative contributions of individual cargo components within 214-EV to the observed therapeutic effects. Moreover, identification of putative miR-214/199a target genes in recipient sciatic nerves and SCs are warranted.

To strengthen potential translational values of 214-EVs in DPN, additional investigations are required, including dose-response and therapeutic window studies, evaluation of long-term therapeutic effects and durability, as well as studies using female mice.

## Conclusion

In conclusion, our study provides the first evidence that engineered miR-214 enriched SC-EVs amplify the therapeutic efficacy of SC-EV for DPN in HFD/STZ-induced T2D mice. The promising results of this cutting-edge technology suggest significant therapeutic potential for patients with DPN.

## Data Availability

The original contributions presented in this study are included in this article/Supplementary material, further inquiries can be directed to the corresponding author.
