# Supplementary material for: Engineered miR-214 enriched Schwann cell-derived extracellular vesicles amplify therapeutic efficacy for peripheral neuropathy in T2D mice
Source: Front Cell Neurosci. 2025 Aug 29;19:1649830. doi: 10.3389/fncel.2025.1649830 (PMC12426067; doi:10.3389/fncel.2025.1649830)
Supplement: Supplementary file 1 [file Table_1.docx]

**Supplemental Table 1. Antibodies used for Western Blots**

| **Antibody name** | **Company and catalog number** | **concentration** |
| --- | --- | --- |
| Anti-CD9 | Abcam, ab92726 | 1:500 |
| Anti-CD63 | Santa Cruz, sc5375 | 1:250 |
| Anti-HSP70 | Abcam, ab31010 | 1:500 |
| Anti-Alix | Cell Signaling, 2171 | 1:500 |
| Anti-TSG101 | Thermofisher, MA5-37764 | 1:500 |
| Anti-Calnexin | Abcam, ab223052 | 1:500 |
| Anti-PTEN | Cell Signaling, 9559 | 1:500 |
| Anti-cJUN | Cell Signaling,9165 | 1:500 |
| Anti-pNFkB | Cell Signaling,3033 | 1:500 |
| Anti-TLR4 | Novus Biologicals, 56566SS | 1:500 |
| Anti-β actin | Abcam, ab6276 | 1:1000 |
